# Quantitative Attribute Analyses with Ground Penetrating Radar for Infrastructure Assessments and Structural Health Monitoring

**DOI:** 10.3390/s19071637

**Published:** 2019-04-05

**Authors:** Isabel Morris, Hiba Abdel-Jaber, Branko Glisic

**Affiliations:** Department of Civil and Environmental Engineering, Princeton University, Princeton, NJ 08540, USA; h.abdeljaber@gmail.com (H.A.-J.); bglisic@princeton.edu (B.G.)

**Keywords:** GPR, material properties, attribute analysis, concrete, dielectric constant, Streicker Bridge, infrastructure assessment

## Abstract

In civil structures and infrastructure, assessing true performance and characterizing unusual structural behaviors can help avoid severe structural problems. To further refine or validate the conclusions from structural health monitoring (SHM) analyses, nondestructive evaluation or techniques (NDE or NDT) can be applied in conjunction with SHM approaches. Ground penetrating radar (GPR) is an NDT that has been used to investigate defects and internal features in concrete structures, but is not commonly used to assess mechanical properties for the purposes of SHM. As a preliminary investigation of the effectiveness of attribute analysis techniques, a GPR survey was conducted on Streicker Bridge (a pedestrian bridge on Princeton University campus with embedded fiber-optic strain and temperature sensors). The bridge was constructed in two phases, where different curing conditions produced different material properties (compressive strength of 51 MPa and 59 MPa). Both standard processing techniques and attribute analysis techniques were employed to interpret GPR reflections in each phase of construction to identify construction elements and to compare the attribute signatures of different strength concretes.Though this study presents primarily relative differences, the sensitivity of these attributes to material property differences is confirmed. This validates SHM studies of the bridge and indicates the potential of the attribute analysis method for material characterization, especially as a compliment to other SHM and NDE techniques.

## 1. Introduction

### 1.1. Structural Health Monitoring + Nondestructive Evaluations

As the world’s infrastructure ages, structural health monitoring (SHM) is a process encompassing a collection of methods and tools with which the performance and safety of these structures can be assessed. These methods and tools are important in estimating the true capacity of a structure (altered by damage or age) and understanding unusual behaviors, which can prevent costly repairs and replacements as the structure deteriorates [1]. SHM methods are based on monitoring a variety of parameters, including strain, temperature, and vibration. SHM tools monitor these parameters for a variety of objectives [1]. SHM projects can be deployed on existing structures (e.g., [2,3]), or installed during the construction process to provide important reference state and early age information. SHM goals, whether damage or performance, can often be met with a combination of nondestructive tests and SHM techniques. In this work, performance of an existing pedestrian bridge is further assessed and characterized with ground penetrating radar (GPR). Ground penetrating radar falls into the category of nondestructive testing techniques, where a material is investigated without using invasive or destructive methods such as removing and testing samples. Nondestructive methods vary greatly in scale and context of application, and in the information gained. Most are based on the principles of wave propagation using sound waves, ultrasonic waves, or electromagnetic waves of almost any frequency. Often these methods are aimed at determining or assessing the internal condition, composition, and structure of a material. While both NDT and SHM aim to assess structural health, condition, and performance, there are key differences between them. NDT is commonly performed ad-hoc, such as when a particular problem is identified or a component is being investigated in a relatively short period of time (e.g., hours to days), whereas SHM is ideally performed continuously (and often also applied ad-hoc) for longer periods of time over the life of the structure. The conditions during a NDT are usually controlled or well defined, while SHM conditions are not controlled and often relatively uncertain. In both NDT and SHM, most techniques for data collection can be automatically applied after the initial set up, but in NDT they are commonly applied manually to the structure. From the above discussion, it is apparent that the boundary between NDE and SHM is rather fuzzy—ad-hoc, short-term measurements, performed in semi-controlled conditions can be considered as NDE, SHM, or both. Commonly, NDE and SHM share Supplementary Information with each other. For example, a NDT can be used to estimate a parameter used in SHM, or damage detected by SHM can trigger additional NDT investigations. GPR, as applied in this project, falls into this intersection between NDE and SHM: while it is an established NDT, this project evaluates its applicability as informational tool for SHM.

### 1.2. Ground Penetrating Radar

Ground penetrating radar (GPR) is a proven method for non-invasive inspection of subsurface features. GPR is a technique by which low frequency electromagnetic signal pulses are emitted at the surface of a medium (e.g., ground, concrete) and the reflected signals are recorded. Each recording is a timeseries of the strength of the incident electromagnetic (EM) field (measured indirectly by the antenna as an induced voltage), which can be used to map and localize internal features (e.g., cracks, inclusions). The transmitted signal is reflected at interfaces or features where there is a change in the electromagnetic properties of the two media (relative dielectric constant, electrical conductivity, and magnetic permeability). When this reflection arrives back at the antenna, its arrival time (a two-way travel time) and relative strength is recorded. A timeseries recording of the reflection strength (as an incident electric field) is called a trace. As the GPR is pushed or pulled along a transect, traces are recorded at discrete spatial locations. The transect (or radargram) is a matrix of data whose columns represent individual traces. Using the average velocity or dielectric of the medium, the travel time can estimate the depth to reflections, which transforms the transect data into useful and spatially accurate maps of the subsurface from a GPR perspective. For a more detailed discussion of the principles and methods of GPR surveys, the interested reader is referred to the many excellent books on the subject (e.g., [4,5,6]).

Monostatic antennas are most commonly used for underground utility location, concrete inspection, and other civil structure applications, which have a fixed separation between the transmitter and receiver. The size of the antenna is determined by the center frequency of the pulses they emit; civil applications regarding concrete often use high frequency antennas with a frequency ranging from around 900 MHz to 2.6 GHz. These high frequency signals have small wavelengths, which allow them to resolve smaller targets (i.e., rebar, delamination) but have limited penetration depth (up to 1 m with a 900 MHz antenna in ideal conditions). GPR antennas in the medium frequency range (270–900 MHz) are used widely in applications ranging from utility location to archaeology. Antennas with frequencies as low as 30 MHz are used to investigate geological strata and glaciers. This enormous range of GPR applications reflects the bounty of different scenarios and environments where GPR proves useful.

In civil engineering, GPR is applied to map and locate utilities [7,8,9], monitor and inspect concrete structures for defects and anomalies in the service of civil infrastructure SHM and life-cycle management (e.g., [10]). Specifically, GPR has been used for damage identification in different ages and mixes of concrete [11], corrosion detection and prevention [12,13], robust estimates of pavement thickness [14,15,16] and identification of voids and other defects in civil structures [10,17]. Some work is being done on estimating and tracking the evolution of volumetric water content and dielectric properties, but mainly in the interest of understanding early age concrete hydration [18,19,20]. Specifically, the role of water in both the electromagnetic properties and the physical properties of concrete has been highlighted as an important factor to consider in the field of nondestructive material characterization using GPR [8,18,21,22]. This mirrors the relationships used in remote sensing and noninvasive estimation of soil moisture by dielectric constant [23,24,25,26,27]. Applying these relationships to concrete indicates that these attributes are also related to the physical properties (i.e., pore water content and related measures of porosity) of construction materials, as in [28,29]. These physical properties are, in turn, related to the mechanical or engineering properties of the materials (like compressive strength or Young’s Modulus) by established methods like ultrasonic pulse velocity testing (e.g., [30,31]). However, GPR is not yet widely used in civil engineering materials characterization or to non-destructively estimate physical properties of interest like porosity or density, or mechanical properties for the purposes of SHM.

### 1.3. GPR Attribute Analysis

In addition to the qualitative interpretations of GPR data described above, attribute analysis is another field of GPR data analysis that can be applied to learn more from conventional GPR data. This quantitative analysis method is used extensively in seismic data processing and has been applied with moderate qualitative success to GPR data. GPR attribute analyses have been used to successfully differentiate between plastic, concrete and metal [32,33], perform transient analysis of contamination [34], estimate concrete hydration and water content (e.g., [18]), estimate soil dielectrics [35], and interpret subtle archaeological features [36,37]. The method generally includes performing computations or transforming the numerical data from a GPR scan and using the results to qualitatively or quantitatively interpret the data [2,3]. The attributes commonly used for these investigations in a civil engineering context include amplitude and frequency-based attributes such as energy [3,32,33], image processing methods [37], and transient attributes [34]. However, none of these studies have asked questions about how the physical and mechanical properties of materials are implicated in GPR data. These properties, especially when combined with health assessments of the individual components of a structure, are directly indicative of the performance and therefore the safety of the structure. Hence, the aim of this work is to explore the capacity of amplitude-based attributes and directly available electromagnetic attributes to directly capture variations in material properties and to locate and map internal construction elements in their actual locations. A post-tensioned reinforced concrete pedestrian bridge with two different construction phases and concrete strengths located on the campus of Princeton University was used as the case study and proof of concept of attribute sensitivity to material properties. This work is inspired by a number of relationships indicated in the cited literature and the authors’ ongoing work exploring direct relationships between the electromagnetic GPR attributes and the physical properties of construction materials [2,3]. These results indicate the promise of attribute analysis for independent material property characterization and the need for further experimental work to develop reference-free frameworks using GPR attribute analysis.

## 2. Materials and Methods

### 2.1. Streicker Bridge

**Figure 1:**
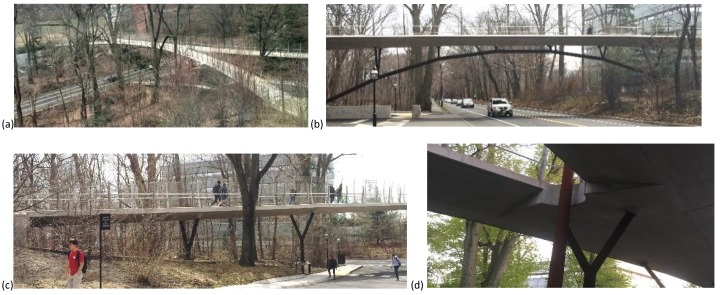
Streicker Bridge: (**a**) near-plan view: main span and approach legs, (**b**) main span: deck stiffened arch, (**c**) monitored SE leg: curved continuous girder, and (**d**) springing of main span.

Streicker Bridge provides vital pedestrian passage over Washington Road, which runs through the south end of the campus of Princeton University (Figure 1). It serves the busy connection between science and lab buildings for a number of departments and connects athletic fields to the auxiliary facilities. The bridge deck is formed by a central arch which splits into two approach spans on either side of the road, in plan forming a curved x-shape. The four approach legs and main span are supported by y-shaped columns, with the main span (35 m) functioning as a deck-stiffened arch and the legs as curved continuous girders for a total length of around 104 m (Figure 1b,c). The deck is constructed of post-tensioned high-performance concrete with a specified 28-day strength of 41.4 MPa (6000 psi); the steel columns are made of concrete filled weathering steel [2,3,38].

**Figure 2:**
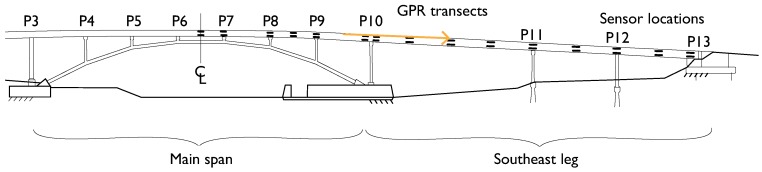
Elevation view of locations of parallel sensors along the main span and southeast leg of Streicker Bridge.

The main span and three of the approach legs were cast and constructed in August 2009. A construction delay forced the remaining SE leg of the bridge to be constructed later, during October 2009. Although the same nominal properties of the concrete were specified, compressive tests from the concrete in each phase indicate that the different batches and curing conditions produced stronger concrete in the SE leg. The specified 28 day compressive strength tests on reserved cylinders indicate that the first phase has an average compressive strength of 51 MPa (7390 psi) and the SE leg has 59 MPa (8663 psi) [1,38]. Though these strengths have likely changed as the bridge ages and are no longer the true strengths, their relative difference remains (refer to [38] and the references listed below). In addition, the bridge was partially instrumented with a SHM system consisting of parallel sets of long-gauge fiber optic strain and temperature sensors embedded in the deck. The SE leg and adjacent half of the main span are instrumented as shown in Figure 2. These sensors have generated several important results, including the detection and characterization of cracks [39,40], assessment of prestressing forces [41], temperature driven SHM using thermal gradients [42,43], and other strain-based parameters related to the stiffness of the SE leg and main span [38,44]. Detailed presentation of the monitoring project and early findings can be found in [1,38]. The SHM system based on fiber-optic sensors was used to determine the average elastic modulus in the two monitored regions of the bridge: 36 GPa in the southeast leg and 33 GPa in the main span [38]. While the NE leg considered in this work is not instrumented or directly monitored by SHM, it was constructed at the same time as the main span, thus is it reasonable to assume similar material properties in the NE leg and main span. The GPR results are consistent with this assumption.

### 2.2. Data Collection

**Figure 3:**
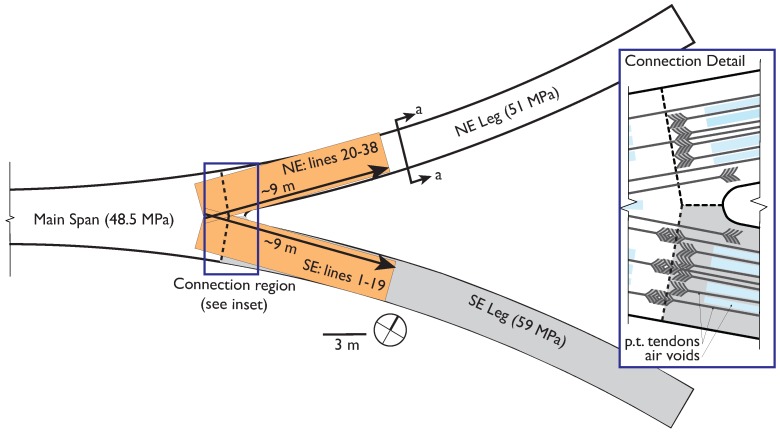
Plan view of scanned areas of Streicker Bridge with strength and detail of connection region.

**Figure 4:**
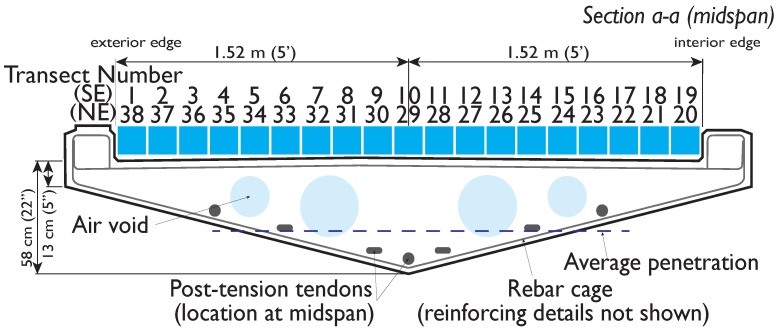
Section a–a taken between columns at midspan (post-tension tendons at their lowest point) showing location of GPR scans relative to internal features. The detailed reinforcing in the section is not included for clarity; only the contour of rebar cage is shown. Transects run longitudinally from the main span down the SE leg.

The GPR survey of Streicker Bridge was conducted on one day in November, 2016 with a GSSI StructureScan 2.6 GHz antenna (see Supplementary Materials). The focus was on comparing the northeast (NE) leg and southeast (SE) leg, which encompass the concretes with known material properties. In the days leading up to the survey, weather conditions were dry and clear, with no recent significant precipitation events or surface moisture on the bridge deck. For a traditional GPR investigation, 2.6 GHz frequency offered a good balance of high resolution with a reasonable concrete penetration depth of up to 40 cm. The unit is commonly used in civil infrastructure applications, with the advantages being a live display of the radargrams and small, easily maneuvered antenna configuration. The device itself is approximately 15 cm × 23 cm × 18 cm and is rolled along the transect directly on the structure’s surface. Both longitudinal and transverse scans of the bridge deck were collected. The complete raw GPR scans of the NE and SE legs, are available as Supplementary Materials. The longitudinal transects, which are the focus of this work, were collected at 15 cm line spacing and have a nominal length of 8 m (Figure 3 and Figure 4). The longitudinal transects are straight but their length varies because of the deck’s curvature. The location of the internal features of the deck (intentional voids, reinforcing, post-tension tendons) are shown in Figure 3 and Figure 4, indicating the tendon locations at mid-span relative to the approximate trajectory of the longitudinal transects (Figure 4).

### 2.3. Data Processing

The high electromagnetic contrast between materials in this survey (air, concrete, and metal) produce data which is relatively clear and requires minimal processing, despite high attenuation of GPR signals in concrete. A standard set of basic filters were applied to the data. These filters were selected so that the relative amplitude of the reflections is preserved rather than the absolute amplitude, as the absolute amplitude is highly variable with respect to the electromagnetic properties of the material and to antenna ground coupling, surface roughness, and other external factors [34]. Most processing was done using a combination of Matlab and Radan [45]. A time-zero adjustment was applied to remove the initial reflection of the concrete/air interface and then the data were dewowed. Dewow removed low frequency noise from the data (including reflections from inside the antenna itself). Amplitude attributes were computed on a truncated portion of these data; instantaneous attributes were computed on the same data before it was dewowed. The data were truncated to remove the connection between approach spans and main span and reflections from below the shallowest depth of the deck. Truncating the data in this way allows all the transects to be compared regardless of the depth of the deck at that location. For visual interpretation, the complete scans (untruncated) were dewowed and further processed by applying gain (automatic gain control, with 2 ns window) and mean background removal to aid in visual interpretation of features below the prominent upper rebar cage. Gaining is a process of amplitude recovery that compensates for signal attenuation with depth. Even though the absolute (raw) amplitudes were not retained after gaining, the relative amplitude of the reflections is preserved. Both standard visual interpretation and quantitative attribute analyses were carried out on the data set and interpreted in conjunction with construction documents. The trace amplitudes were recorded as an induced voltage from the incident electric field on the antenna and, as such, arbitrarily depend on the particular antenna being used [4]. The reflection amplitude attributes are presented here as unitless; propagation attributes like total energy were normalized and unitless.

### 2.4. Attribute Analyses

An analysis of the amplitude-based attributes was performed on lightly processed data (time-zero adjustment, dewow, and truncation), summarized in Table 1. Two reflection attributes and two propagation attributes were calculated. Reflection attributes quantitatively compared individual extreme reflection events, like the strength of a rebar reflection or the ground wave, in two different kinds of concrete. Because this investigation was into the relative material properties of concrete, which can be considered relatively homogeneous with discrete inclusions, both reflection and propagation attributes are relevant. The triangular (NE and SE leg) and trapezoidal (main span) bottom face of the deck caused some transects to include the reflection of the bottom surface of the deck, while others did not reach the lower surface (Figure 4). Before calculating the amplitude attributes, the data were truncated so that they each had the same number of samples and had only propagated in the deck of the bridge. Additionally, this step ensured that a similar configuration of the rebar cage, concrete, and other internal components is scanned in each transect. Each reflection and propagation attribute was calculated for all traces in the transects and then compared laterally across the deck and between the legs of the bridge. The two reflection attributes presented here are the maximum amplitude and maximum absolute amplitude; the intensity (squared amplitude) was also examined, though the results followed the same trend as the absolute amplitude attributes and are omitted. Propagation attributes quantify the entire time history of the trace and include average behaviors and trends in addition to the individual reflection events. Propagation attributes include the total area of the trace, total area of the absolute trace, and total energy of the trace. The total area of the trace was not reported, presenting the same trends as the other propagation attributes.. Total energy of the trace was calculated by integrating the intensity of the trace ([33], Equation (1)):

(1) $$E_{t}=\int_{t_{1}}^{t_{2}}A{\left(t\right)}^{2}dt$$

where $E_{t}$ is the total energy, $t_{1}$ and $t_{2}$ are the start and end times included in the calculation (the same times for each trace considered), and *A* is the trace amplitude. This can also be normalized by the length of the trace to arrive at a measure analogous to signal power, but here are presented as arbitrary energies of identical length traces.

In addition to these time domain amplitude attributes, electromagnetic attributes and instantaneous attributes were also computed. For electromagnetic attributes, the relative dielectric constant is of interest because it is directly related to porosity and water content in natural stones and concrete [18,28]. The electromagnetic attributes include the average velocity and dielectric constant (ε), which are directly related (Equation (2)) and were computed using Radan 7’s geometric hyperbola fitting and Equation (2) [45]:

(2) $$v=\frac{c}{\sqrt{\varepsilon }},$$

where *v* is the group electromagnetic velocity, *c* is the speed of light, and ε is the relative dielectric constant. The hyperbolic shape of the reflection produced by perpendicularly scanning a piece of rebar is a function only of the depth, velocity, and spread of the hyperbola. These reflections (sometimes called diffractions) were used to estimate the average dielectric and velocity of the concrete from at least five hyperbolae in each transect and each leg. The steepest hyperbolae were chosen to minimize error and include those rebar reflections which were most perpendicular to the transect [4]. The geometric hyperbola fitting technique is manual, performed in the Radan software package by pattern matching. The technique is explicitly sensitive to the electromagnetic properties in Equation (2), but is also sensitive to small surface variations, the angle at which a transect crosses the rebar, and the software’s visual limitations. The velocities estimated by hyperbola fitting were verified using travel times and the known depth to some of the internal features in the deck section (i.e., ground truth). The geometric fitting procedure was used in this work because of its high performance in capturing relative difference between two concretes from a large number of sample hyperbolas.

Instantaneous attributes included in this work are based on the Hilbert transform of the individual raw traces, where instantaneous amplitude (α(t)), instantaneous phase (φ(t)), and instantaneous frequency ($\varphi^{\prime }\left(t\right)$) are readily available in the analytic signal. A frequency domain analysis was performed on the truncated data using the discrete-time Fourier transform to estimate the centroid frequency of the signal in each transect and leg of the bridge. The centroid frequency presented here is a weighted average of all the frequencies present in each analytical trace. For each transect, the mode of the centroid frequency in each trace was found and then the average in each leg was compared. Representative distributions of centroid frequencies in two pairs of transects are also presented. These electromagnetic and instantaneous attributes vary based on both frequency and amplitude, and are therefore especially useful in exploring the differences between the dispersive electromagnetic properties of the concrete in the NE and SE legs of the bridge, and how those can be related to the physical properties. The instantaneous attributes (frequency, amplitude, and phase) were calculated for the entire data set, meaning that these transects were not clipped and include the reflection from the lower surface of the deck. This was done to investigate the robustness of the method to the variation in deck thickness.

## 3. Results

### 3.1. Qualitative Analysis

**Figure 5:**
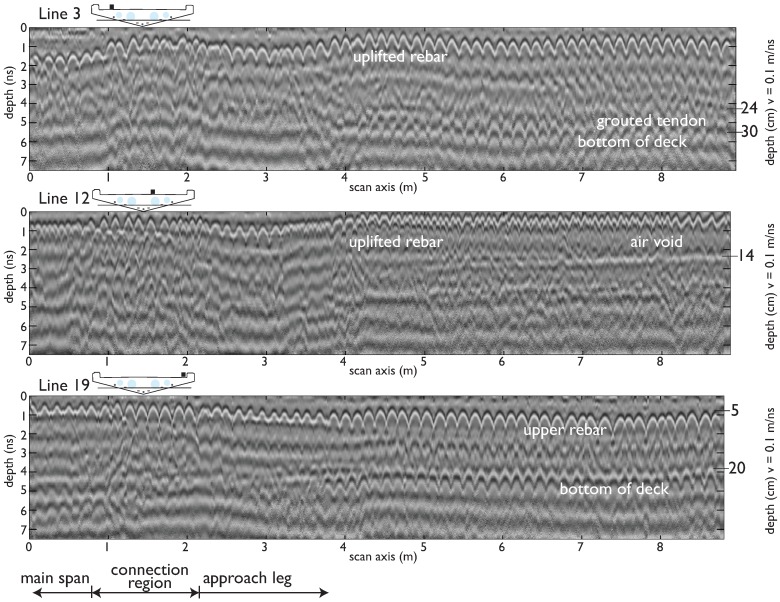
Processed unmigrated transects 3, 12, and 19 (SE leg) showing the internal features of the deck, with depths confirmed in construction documents using a nominal velocity of 0.1 m/ns. Note the lower rebar cage at the bottom of the deck and uplift of the upper rebar cage from the air voids.

Qualitative visual analysis of the GPR scans frames quantitative analyses of the GPR data and positively identifies each feature present in construction documents and physical inspection in the bridge deck, including air voids, deck surfaces, and reinforcing bars (Figure 5, [38]). The lower surface of the deck and air voids are clearly visible in shallower transects of the deck, appearing as a strong air-concrete interface because of the high electromagnetic contrast between concrete and air, which has high reflectivity (Figure 5). The lower surface of the deck was only visible for longitudinal scans of the bridge that are located near the edges, where the deck has a smaller depth (e.g., lines 3 and 19). This is a function of the antenna frequency and penetration depth, which is, on average, 38 cm (less than the full 58 cm thickness of the deck, indicated in Figure 4) and was validated with the geometric properties of the the deck as designed. The locations of the air voids in the deck section, created by embedded PVC tubes, were consistent with construction plans for the bridge [38]. GPR scans reveal vertical camber of the air voids and rebar cage relative to the top of the deck surface (lines 3 and 12 vs. line 19), which was not indicated in the designs. When the bridge was constructed, the voids were not secured properly, so in some locations buoyant forces propelled them towards the deck surface before the concrete hardened. The rebar uplift validated in GPR scans indicates areas of insufficient concrete cover and high likelihood of rebar exposure on the upper surface of the bridge itself (lines 3, 12, Figure 5, [38]). Maintenance of the bridge reflects this risk, employing special deicing agents to minimize corrosion of upper rebar. As evidence of this uplift, there was rebar surfacing in the main span of the bridge and GPR scans confirm the trajectory of the rebar cage and air voids in GPR scans.

Longitudinal post-tension cables commonly have a “draped” profile, where the continuous tendons are located in the region of the cross section which is likely to experience the most tensile stresses, i.e., at the center of the span of a continuous beam, near the bottom and near the top as they pass over column supports [38]. During construction, these tendons are laid freely in conduits before the concrete is poured. After the concrete has developed sufficient strength, tension is applied to the tendons and they are grouted in place (hence, post-tensioned). In relation to GPR, this construction weakens the reflection strength of the tendons because the combination of conduit, grout, and metal tendon creates a series of smaller interfaces with lower contrast than the sharp interface between concrete and air. Despite this weaker reflection and small size of the tendons, the draped profile actually aids in identifying the reflection of the tendons along the deck in the relevant GPR scan and confirmed in construction documents. One tendon was visible in line 3 (Figure 5). Visual differentiation between internal components was supported by confirming the depth of the feature in construction documents using a nominal velocity of 0.1 m/ns (the estimated velocity using hyperbola fitting is around 0.118 m/ns in the SE leg).

At the connection between the main span and the SE leg, the deck changes cross section to accommodate the main span (Figure 1d). The connection region appearred slightly larger in the scan than in construction documents (at least 1 m vs. 80 cm), which was indicative of the additional measures to ensure continuity between the main span and SE leg. These include reinforcing and the lack of a continuous tendon through the leg and main span (Figure 3, [38]). GPR scans showed that the overlapping rebar connecting the leg to main span extends over a wider area in the SE leg than in the NE leg. For structural health monitoring aims, visual interpretation of GPR scans can be supplemented with quantitative analyses to create a complete and accurate model of the various components of a structure as built, which includes all differences from design (e.g., uplifting of rebars, etc.) [38]. This model was essential to monitoring the performance of a structure and establishing the reference behavior from which variations can be found.

### 3.2. Quantitative Analysis

**Figure 6:**
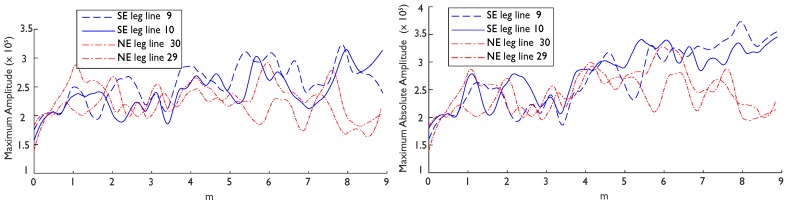
Reflection attributes: maximum amplitude (**left**) and maximum absolute amplitude (**right**) for two representative transects in the center of the deck, showing increased values for the southeast (SE) leg (blue solid and large dash) to the right side of the connection between the two construction phases.

**Figure 7:**
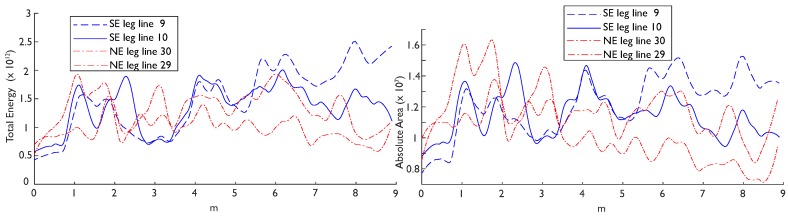
Propagation attributes (smoothed): total energy (**left**) and absolute area (**right**) of the two construction phases, the SE leg (blue solid and large dash) and NE leg (red dotted), show the distinction between the two concrete mixes for two representative pairs of transects at the center of the deck, more pronounced in the energy attribute than absolute area.

The reflection attributes from each leg of Streicker Bridge indicate that, overall, GPR can identify a difference between the material properties in the two legs of the bridge; the stronger concrete in the SE leg is indicated by higher average amplitudes in the GPR signals (Figure 6 and Figure 7, Table 2). In these figures, the attributes were smoothed (with a moving average that divides the data into ten sections) and plotted along the full transect (including a portion of the main span and connection region from 0–3 m) before being truncated and presented in Table 2. The attributes for two adjacent transects in the center of the deck are plotted and tabulated to indicate the variation in one leg; Table 2 additionally contains transects located on the edge of the deck. Of these computationally simple results, both maximum amplitude and maximum absolute amplitude followed the same general trend of larger amplitudes overall in the SE leg, excepting transects that contain the strong reflections of the air voids (e.g., Table 2, Figure 6: lines 16 and 23). In the NE leg, the concrete itself had a higher dielectric constant, thus increasing the contrast between the air void (dielectric = 1) and the concrete (dielectric ≈ 7) and increasing the strength of the reflection. This resulting implication was that the attributes were not robust to changes in the composition of the deck (including reflections from voids, reinforcing, and other materials), but were nominally sensitive to relative material strengths.

In the propagation and reflection attributes plotted for a whole transect, the difference between concretes became more pronounced further down the leg (to the right, Figure 6 and Figure 7). Early in the transects (to the left), the only difference between the two legs was at the connection to the SE leg. After about 4 m along the transect (roughly where the air voids in the legs begin), the SE leg attributes became increasingly larger than the NE leg attributes. These trends were also observed in the propagation attributes (total energy and absolute area), which have highly consistent results with each other (Figure 7). These attributes consider the whole trace and not just a single reflection event, which lends them to analysis of a structure which has a highly variable composition along one longitudinal direction. Mathematically, the selected attributes consider both strong positive reflections and all reflections without regard for polarity. This combination of attributes indicated that there was a difference between the reflection strength in both a positive and absolute sense. However, the difference between the two concretes was not significant using basic statistical measures (i.e., 2 sample t-tests); there was a significant difference in each attribute between legs, but each transect was also significantly different than the others in the same leg (i.e., there is less than a 5% probability that they come from the same distribution). In two pairs of lines in the center of the deck (9/10 and 30/29), the attributes had very high spread (interquartile ranges an average of 81% of the mean). Towards the edges of the deck (lines 16/23 and 19/20), there was less spread (interquartile ranges an average of 72% of the mean), but also a reversed trend in the mean attributes (Table 2). Hence, future research is needed to address this challenging variation within the same material (but different internal structure) before exploring how the attributes vary between the construction phases.

Similar trends were presented in a targeted analysis of the instantaneous attributes by comparing three representative pairs of transects, each of which were located at the same relative point along the cross section of the deck (Figure 4, Table 3). The same relative amplitude trends were seen in the instantaneous attributes (such as average instantaneous amplitude) as in the reflection and propagation attributes, where the stronger (SE leg) concrete had generally higher values. The average instantaneous frequency was lower in the SE leg than the NE leg, paralleling both the phase attribute and the centroid frequency trends. Note that, similar to the amplitude attributes, the trend was not followed by the pair of transects at the edge of the deck (transects 19 and 20). In these attributes, the edge effect was dominated by using the entire traces and not the truncated data. Not surprisingly, the instantaneous attributes, including frequency, were not robust to the variable deck composition and cross section.

Comparing the electromagnetic properties as attributes confirms the trends present in the other attributes and provides additional insight into the mechanisms behind the relationship between GPR attributes and material properties. The SE leg has a lower mean dielectric constant (ε) than the weaker NE leg: 6.65 vs. 6.99 (*p* = 0.097) (Table 4). This corresponds to higher velocities in the stronger SE leg; in the SE leg, there is a smaller spread of data and fewer outliers than the NE leg. Recall that the dielectric constant was determined by manual geometric hyperbola fitting for at least five hyperbola in each transect. When the nature of this test was considered, there was a more significant difference in mode between the dielectric in each leg (*p* = 0.0028) than other statistical measures. The mechanisms behind the concrete properties effect on the signal are still under investigation [24,26,46], though these results are consistent with findings in other works related to the transition from free water to bound water (e.g., [18]). Ultrasonic results strongly supported higher material strength correlated with lower porosity and higher density [31,47]; in [18], the authors hypothesize that there is a combination of mechanisms is at work. Higher porosity (higher dielectric) caused more scattering of the GPR signal, resulting in smaller amplitudes and corresponding to slower velocities; when there was more free water which can be polarized, often in a weaker and more porous concrete, the dielectric constant was larger (e.g., [18]).

**Figure 8:**
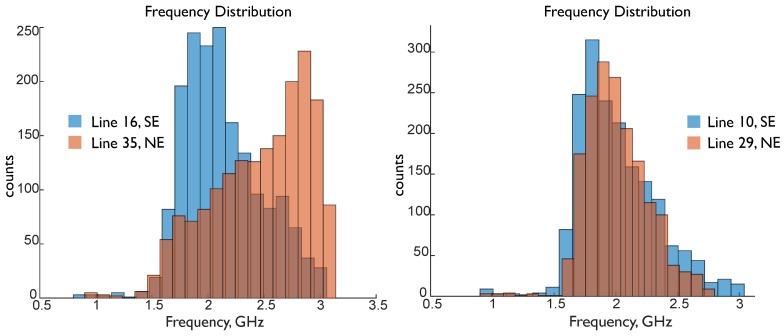
Frequency distribution of representative transect pairs 16 and 35 (**left**), and 10 and 29 (**right**), with a lower overall centroid frequency in the SE leg.

The centroid frequency comparisons are aligned with the dielectric constant hypotheses regarding porosity. The average and more of the centroid frequency for the traces in at least 2/3 of the scan pairs (with corresponding locations on the NE and SE legs) was higher in the NE leg than the SE leg, as shown in the two representative frequency distributions in Figure 8. The average of the mode of the centroid frequencies for all transects in the NE leg was 1.33 GHz and 1.06 GHz in the SE leg. A lower centroid frequency indicates that there was higher attenuation of the signal, especially of the high frequency components. Higher water content in concrete was accompanied with higher porosity and lower strengths; higher water content was also associated with less attenuation. That is, as the signal propagates through weaker concrete, the dominant attenuation mechanisms were scattering and polarization of free water (e.g., [18]); in stronger concrete, there were smaller and fewer pores, as well as less free water, so the dominant attenuation mechanism was the signal propagating through the material itself. This is consistent with higher amplitude-based attributes in stronger concrete, resulting from the stronger reflections of a less scattered and lower dominant frequency signal (SE leg).

## 4. Discussion

**Figure 9:**
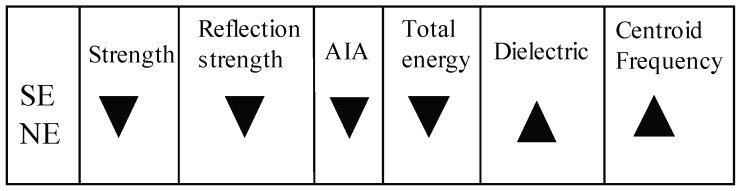
Summary of relative attributes and properties, where arrow widens toward the larger attribute.

This paper presents a high frequency GPR survey of a reinforced post-tensioned concrete pedestrian bridge and associated attribute analyses with the principal aim to explore the capabilities of these attributes to asses physical and mechanical properties of materials. Additional aims specific to the bridge include assessment of the internal structure and composition of the deck and the differences between the two construction phases. The location and presence of each feature included in the construction drawings (e.g., air voids, post-tension tendons) can be identified in the GPR data, as can important deviations from those plans (e.g., details of SE leg connection, rebar uplift). GPR reflections at air-concrete interfaces are stronger than the reflections at embedded and grouted post-tension tendons and these attributes can be used to readily distinguish between features based on material identity. Stronger reflections are produced in stronger concrete (higher amplitudes, slower travel, and higher attenuation for a lower centroid frequency Figure 9). Though the present strength of these two phases is unknown, the relative difference is still present (as confirmed by strain and temperature based SHM of the bridge). This specific application of GPR attribute analysis on the bridge serves as a proof of concept and motivates development of a reference-free framework for material property characterization using GPR. With only two unknown sample strengths, it is not yet possible to estimate the present strength of the concrete, but work is ongoing to develop a more universal technique.

It was found that the electromagnetic attributes have the potential to differentiate materials with different mechanical properties, as in soil moisture and petrology. Comparing amplitude-based attributes, instantaneous attributes, and electromagnetic attributes between the concretes with two different strengths establishes trends in the relationship between relative material properties and GPR attributes. Perhaps the most compelling result of this work is that the maximum absolute amplitude, the simplest attribute, is the clearest indication of the relative difference between the two concretes. The relationship between attributes and relative strength in the two legs of the bridge agrees with similar works relating dielectric to soil moisture and some other natural building materials [24,26,27,28,29], and to the relationship between material strength and porosity established with ultrasonic methods (e.g., [30,31,48]). Thus, the application on Streicker Bridge serves as a motivation and proof of concept to pursue more universal GPR techniques that can estimate material properties directly. As with Streicker Bridge, these results and analyses can be incorporated into the wider monitoring project and used for future studies. This includes verifying the different behaviors in certain regions of the deck, aiding estimation of parameters relevant to creating improved SHM projects (such as coefficient of thermal expansion or density), and providing a more complete or updated reference configuration for the structure. GPR helps better describe the composition and structure of the bridge, which can be used in conjunction with continued SHM monitoring and established abilities of GPR, including tracking continued behavior of the connection, the monitored spans, concrete cover and rebar corrosion risk, and the bridge as a whole. That is, a GPR survey can be collected on any piece of infrastructure to gain specific information about the state of health. Whether the GPR survey is in conjunction with another SHM sensing application or is the only sensing technique applied, continued study of GPR attributes could develop the technique into a robust SHM diagnostic tool. For example, when coupled with strain, temperature, or vibration monitoring, GPR attribute analysis could help validate conclusions about changes in strength or capacity which appear in both techniques. For Streicker Bridge, understanding differences in material properties and behavior of coexisting phases could help prevent and understand damage caused by those differences (i.e., thermal incompatibility), representing a significant benefit to conducting the GPR survey and attribute analysis. More broadly, these results indicate the need for future research and statistical analysis of the relationships between GPR attributes and material properties, as well as continued implementation of GPR in the service of SHM objectives. For the purposes of material property characterization, the results for Streicker Bridge motivate more comprehensive laboratory testing with a controlled sampling of defined materials and a variety of nondestructive techniques; this work is forthcoming. Along with ancillary purposes of feature detection, damage (i.e., crack) localization, decision making, tracking damage and degradation, and validating SHM results, material property characterization provides an additional benefit to performing a GPR survey for broader SHM objectives. GPR scans and attribute analyses provide complimentary information to nondestructive testing and structural health monitoring approaches, validating conclusions, assessing performance, and paving the way for future hybrid investigations using GPR. 

## Figures and Tables

**Table 1:** Summary of studied attributes.

Class	Attribute († Unitless)	Source	Data Preparation	Comparison Level	Expectation
reflection (time domain, amplitude)	† maximum amplitude	max(A(t))	Time zero, dewow, truncation (same number of samples, remove bottom of deck when present)	trace, scan, and leg (mean)	Identify differences between strength of reflector in two concretes with different strengths
	† maximum absolute amplitude	max(|A(t)|)			
	† maximum intensity	$max(A{\left(t\right)}^{2})$			
propagation (time domain, amplitude)	† total energy	$E_{t}=\;\int_{t_{1}}^{t_{2}}A{\left(t\right)}^{2}dt$	Time zero, dewow, truncation (same number of samples, remove bottom of deck when present)	trace, scan, and leg (mean)	Identify differences between amplitudes for a whole trace in two concretes with different strengths
	† total area	$\int_{t_{1}}^{t_{2}}A\left(t\right)dt$			
	† total absolute area	$\int_{t_{1}}^{t_{2}}\left|A\left(t\right)\right|dt$			
electromagnetic attribute	velocity (m/ns) dielectric constant	geometric hyperbola fitting and $v=\frac{c}{\sqrt{\varepsilon }}$	automatic gain control applied	scan, leg (mean)	Assess sensitivity of fundamental EM attributes in differentiating the two concretes with different strength
instantaneous reflection attribute	† instantaneous amplitude	α(t)	Hilbert transform raw truncated trace →$\alpha \left(t\right)e^{i\varphi (t)}$	trace, scan, leg (mean)	Identify differences between non-amplitude attributes in two concretes with different strengths
	instantaneous phase (rad)	φ(t)			
	instantaneous frequency (Hz)	$f=\frac{d\varphi }{dt}$			
frequency attribute	centroid frequency (Hz)	$f_{c}=\;\frac{\sum_{bins}f_{c}\left(i\right)f_{i}}{\sum_{bins}f_{i}}$	Hilbert transform raw truncated trace →$\alpha \left(t\right)e^{i\varphi (t)}$ → FFT to frequency domain	scan, leg (mode)	Identify differences between attenuation of frequency components in two concretes with different strength

**Table 2:** Mean normalized amplitude and propagation attributes (from truncated data) for three pairs of transects: two in the center (10 and 29, 9 and 30) and towards the edge (16, 23).

Transect		Maximum		Maximum Absolute Amplitude		Absolute Area		Total Energy	
**SE**	**NE**	**SE**	**NE**	**SE**	**NE**	**SE**	**NE**	**SE**	**NE**
9	30	1.42	1.26	1.42	1.23	1.64	1.37	2.63	1.96
10	29	1.30	1.00	1.31	1.04	1.32	1.00	1.62	1.00
16	23	1.10	1.38	1.00	1.31	1.35	1.83	1.30	2.46

**Table 3:** Normalized average instantaneous amplitude and frequency for three pairs of transects in each leg, one in the center (10 and 29), at the edge (19 and 38), and midway between an air void and post-tensioned tendon.

Transect		Average Instantaneous Amplitude (Norm.)		Average Instantaneous Frequency (GHz)	
**SE**	**NE**	**SE**	**NE**	**SE**	**NE**
10	29	1.1	1	8.61	8.81
16	23	1.34	1.28	7.67	7.64
19	20	1.12	1.36	7.37	6.69

**Table 4:** Summary of dielectric constant in the stronger southeast (SE) and weaker northeast (NE) leg.

	SE ε	NE ε
mean	6.65	6.99
median	6.53	6.90
mode	6.26	6.90
st. dev.	0.59	0.63

## Supplementary Materials

The following are available online at https://www.mdpi.com/1424-8220/19/7/1637/s1. The complete raw GPR scans of NE and SE legs, collected with GSSI StructureScan HR 2.6 GHz antenna in Spring 2017. Collection settings can be found in the headers of the files.

## Abbreviations

The following abbreviations are used in this manuscript:

GPR	Ground penetrating radar
SHM	Structural Health Monitoring
NDE/NDT	Nondestructive evaluation/nondestructive testing
SE	Southeast
NE	Northeast
EM	electromagnetic
GSSI	Geophysical Survey Systems Inc.
